# Assisted Reproduction Technologies (ART): Impact of Mitochondrial (Dys)function and Antioxidant Therapy

**DOI:** 10.3390/ani15030289

**Published:** 2025-01-21

**Authors:** Filipa C. Ferreira, José Teixeira, Fernando Lidon, Fernando Cagide, Fernanda Borges, Rosa M. L. N. Pereira

**Affiliations:** 1 Unit of Biotechnology and Genetic Resources, National Institute of Agrarian and Veterinary Research, Quinta da Fonte Boa, 2005-424 Vale de Santarém, Portugal; filipa.ferreira@iniav.pt; 2 GeoBioTec—Faculty of Sciences and Technology, New University of Lisbon, Campus da Caparica, 2829-516 Caparica, Portugal; fjl@fct.unl.pt; 3 CNC—Centre for Neurosciences and Cell Biology, University of Coimbra, 3004-504 Coimbra, Portugal; jteixeira@cnc.uc.pt; 4 CIBB, Center for Innovative Biomedicine and Biotechnology, University of Coimbra, 3004-504 Coimbra, Portugal; 5 CIQ-Department of Chemistry and Biochemistry, Faculty of Science, University of Porto, Campo Alegre, 4169-007 Porto, Portugal; fernandocagide@yahoo.es (F.C.); fborges@fc.up.pt (F.B.); 6 CIISA, Faculty of Veterinary Medicine, University of Lisbon, and Associated Laboratory for Animal and Veterinary Science (AL4AnimalS), Av. da Universidade Técnica, 1300-477 Lisboa, Portugal

**Keywords:** fertility, reproduction, gamete, embryos, oxidative stress, antioxidants, mitochondria

## Abstract

In the last decades, major changes in ecosystems related to industrial development and environmental modifications have had a direct impact on mammalian fertility, as well as on biodiversity. It is widely demonstrated that all these changes impair reproductive function. Several studies have connected the increase of reactive oxygen species (ROS) generated in mitochondria to the recently identified decline of fertility due to various factors, including heat stress. The study of antioxidants, and especially of mitochondria targeted antioxidants, has been focused on identifying more efficient and less toxic therapies that could circumvent fertility problems. These antioxidants can be derived from natural compounds in the diet and delivered to the mitochondria in more effective forms, providing a much more natural therapy. The use of mitochondriotropic diet-based antioxidants in assisted reproductive technologies (ART) may be an important way to overcome low fertility, allowing the conservation of animal biodiversity and productivity. This paper provides a concise review of the current state of the art on this topic, with a particular focus on the antioxidants mitoquinone, AntiOxBEN_2_, AntiOxCIN_4_, urolithin A and piperine, and their effects on bovine and other animal species.

## Simple Summary

A direct negative impact of environmental on mammalian fertility, due to industrial development, has made it imperative to find strategies to mitigate the effects. The main causes of fertility decline and ways of trying to increase it are presented and discussed in this review. The particular approach focused on the roles of mitochondria and oxidative stress in this problem and how natural and mitochondriotropic antioxidants can help to solve it.

## 1. Introduction

**Figure 1:**
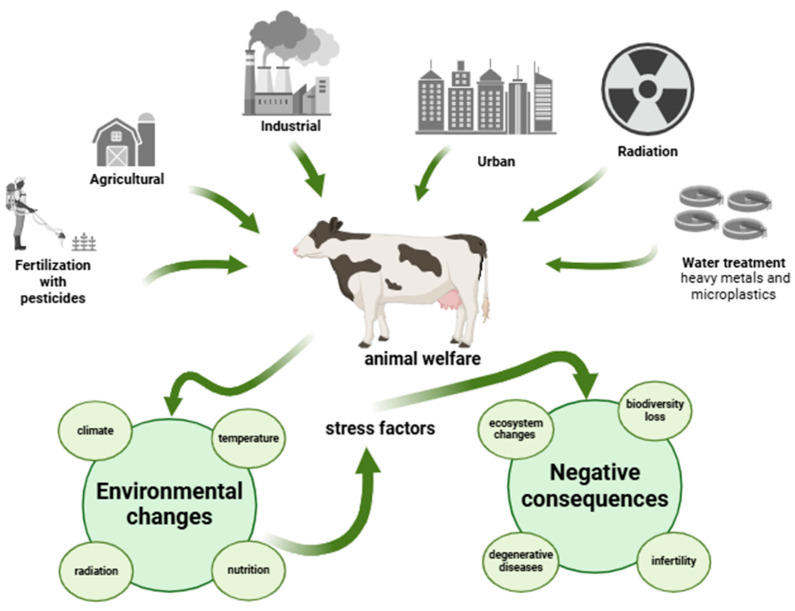
Impact of environmental stress on animal welfare and fertility, as well as on biodiversity (Created in Biorender.com).

In recent years, pollution levels have increased significantly, largely as a consequence of industrialization during the 20th century. Agricultural fertilizers, manure, sewage, heavy metals, plastics, chemicals and industrial discharge are polluting coastal regions, oceans and continental ecosystems. Today, many of these compounds are found in the air, drinking water, soil, food, plants, animals and humans [1,2,3,4]. Poor air quality has become a global problem that is not limited to industrialized cities. All of these changes and exposure to other specific environmental factors, such as temperature, climate, radiation and nutrition, create an environment with increasing stress factors (Figure 1). These factors have a serious impact on the adaptation, reproduction and survival of living organisms by changing the ecosystem and biodiversity, with negative consequences, namely the increase of degenerative diseases and the decrease of fertility [5,6,7]. Several studies have linked the decline in fertility to mitochondrial dysfunction and increased reactive oxygen species (ROS) generated in mitochondria by various stressors [5,8]. In addition, environmental pollution has been shown to impair fertility in all mammalian species [2,9]. Many of these toxic compounds are endocrine disruptors or have endocrine active substances that can damage reproductive performance, which has important social and economic consequences [3,4,8,10].

Several reports since 1969 have shown an increase in fertility problems [11,12,13,14]. The most frequent cause of infertility has been attributed to the low number and quality of germ cells, namely azoospermia and oligospermia in the male or ovulation failure caused by hormonal disorders or behavioral changes in the female. It can also be caused by problems in the pelvic cavities, such as endometriosis, Sertoli cell disorders or obstruction of the genital tract [15,16,17,18]. Infertility can also be linked to other factors, not only genetic or pathological, including issues related to the age of parents, nutritional problems, the environment, stress, and the use of certain drugs [1,4,9,19]. This issue, compounded by factors such as population growth, climate change, socio-economic shifts, economic globalization, and financial constraints, has underscored the urgency of conducting research and developing strategies to manage fertility and maintain biodiversity. These efforts include the preservation of endangered local breeds, which are critical for sustaining ecological balance and genetic diversity [6,20]. It is estimated that a considerable number of species will become extinct in the near future [5].

Environmental changes, often caused by human activity, have repercussions on reproductive effectiveness and on the maintenance of species and subspecies [1,21,22]. Likewise, heat stress, which is closely associated with climate change, can also affect reproductive effectiveness. Different studies have shown that oocytes collected during warmer weather were of lower quality than those collected in winter, especially in extreme heat conditions [8,23], and that spermatogenesis is also affected, leading to low fertilization rates and embryonic development [24]. According to Leroy et al. [14], the fertility of dairy cows has been declining since the mid 1980s. The low quality of oocytes and embryos has been described as the main reason for low birth rates and high embryonic death rates. Low fertility rates are also associated with high production and feeding, and it has been described that balanced diets, with a good supply of protein and energy, are essential for good energy stability and a consequent improvement in gamete quality. For instance, diets rich in starch can improve energy status and, therefore, ovarian activity in the early postpartum period. However, oocyte and embryonic quality can suffer from these diets, and diets with a high protein content can raise ammonia and urea concentrations in the blood, leading to modifications in the female reproductive system [14,25].

It is, therefore, urgently needed to find solutions to mitigate the deleterious effects of environmental and other changes, on fertility and the welfare of animals and humans. This review briefly summarizes the current problems associated with fertility in cattle and other animals, mainly related to stress factors leading to increased oxidative stress, and possible therapeutic approaches using ART and antioxidants, both of natural origin and manipulated to act on the mitochondria.

## 2. Technological Applications to Improve Fertility

The commercialization of bovine embryos is widespread globally, and despite the economic challenges posed by the COVID-19 pandemic, the embryo industry has continued to grow. A deep analysis of data on the international trade of embryos is particularly useful to understand that these technologies have been adopted in one third of all countries, representing more than half of the world’s cattle population [26]. The commercial value of this activity for the meat and milk trade is directly linked to genetic quality and crossbreeding schemes, which can be obtained by using oocytes, semen and embryos from selected donors, as well as through the implementation of reproductive management strategies to improve pregnancy rates. Another advantage is related to the number of “cheap” embryos that can be produced in vitro, compared to those produced in vivo, and used for research [27,28] or infertility treatments, such as embryo transfer for repeat-breeding cows [28,29,30]. Although some limitations associated with the in vitro fertilization technique have been detected, specifically linked to the low rate of produced embryos and some associated problems in the offspring, it is an excellent tool for solving infertility problems and implementing studies related to fertility/infertility [27,31,32].

Extensive research has led to major scientific and technological advances, such as gamete cryopreservation, artificial insemination with sexed semen, cloning, genomic analysis and genome editing, which are helping to improve reproductive efficiency, although epigenetic alterations [33,34,35] and oxidative stress control [36,37,38,39] remain a challenge. It should be noted that ART practiced on different livestock species is a very successful business worldwide and, as such, plays an important role in the development of livestock farming [35,40].

### Assisted Reproductive Technologies (ART)

Artificial insemination (AI) is the oldest technique used in assisted reproduction for both animals and humans. Initially introduced for animals of zootechnical interest in the late 1930s, AI had a strong sanitary focus. However, its large-scale adoption was driven primarily by economic factors. Following World War II, the development of semen cryopreservation greatly facilitated its distribution and expanded its commercial potential, particularly in the dairy industry. Additionally, AI has been applied to address issues related to sexual dysfunction and infertility [41,42,43].

The development of ART has opened new possibilities for the preservation of germplasm and fertility [6]. However, significant challenges remain, including technical limitations in laboratory procedures and escalating costs. To address these issues, concerted and sustained investment in research is essential to enhance these technologies and ensure their accessibility on a global scale [31,44]. Despite great efforts to improve the quality of both oocytes and in vitro produced embryos, the quality of these blastocysts is still inferior to those produced in vivo. These differences can be seen in morphology, metabolism, gene expression and tolerance to cryopreservation, and they persist throughout development [45,46,47].

ART is a term that refers to all in vivo and in vitro treatments or procedures on oocytes and sperm or embryos to establish a pregnancy, and is now widespread throughout the world, even in developing countries [26,44]. ART procedures include superovulation treatments and AI, sexing, in vitro fertilization (IVF), intra cytoplasmic sperm injection (ICSI), embryo transfer and cryopreservation of gametes and embryos [15,28,41]. IVF has been developed for use in humans and cattle, allowing embryo transfer worldwide [48]. Nevertheless, to be successful, IVF requires rigorous control and manipulation of the reproductive cycle to select the greatest number of oocytes and spermatozoa, assessing their quality, as well as the quality of the embryos [47,49]. ICSI, which consists of the microinjection of a single sperm into the cytoplasm of the oocyte, is currently the most widely used method to overcome serious male infertility problems when the IVF method is unsuccessful, making it the most widely used method in human and equine ART [42,49,50]. In horses, ICSI can be used when the number or quality of sperm is low, making it possible to overcome infertility problems in these animals. In addition, oocyte retrieval and ICSI make it possible to manage normally fertile mares and stallions, reproducing and storing embryos of high genetic quality, creating a highly competitive market worldwide [42,43]. However, ICSI raises questions about the health of the offspring, as it goes against natural selection [51], unlike IVF, where insemination itself allows the union of the two gametes in a more natural way. The great success of embryo transfer and IVF rates in cattle have spread their use worldwide; however, ICSI is rarely used in this species [35].

As mentioned previously, ART comprises several stages, which can include ovarian stimulation, semen collection and freezing, IVF or ICSI, and also embryo culture, occasional biopsy, and finally embryo transfer, which involve significant changes to the gametes and/or the embryo environments. It is now known that the early stages of mammalian embryonic development are very sensitive to their microenvironment [52]. Only 70% of oocytes fertilized in vitro reach cleavage after three days of culture, and of these only 20–50% give rise to blastocysts, falling short of development in vivo, even though the technique has been improved over the years [36,48,52]. For instance, pregnancy rates following the transfer of bovine in vitro produced embryos are 10-30% lower than those of embryos developed in vivo [52,53,54].

Deterioration in the quality of oocytes is one of the factors associated with the failure of ART, since their quality is a determining factor of the potential for development of the embryo after fertilization. Ovulation asynchrony and aged oocytes have been reported to be detrimental to the success of AI and embryo production programs in mares, cows and ewes, involving significant economic losses [14,50,55]. Any disturbance in the follicular or other environment affects the maturation of the female gamete and reduces its quality. In vitro maturation models have shown that some of these metabolic alterations reduce the suitability of the oocyte, with repercussions for the development and quality of the embryo. Therefore, a technique to ensure optimal oocyte maturation is needed, as studies indicate that adverse conditions for oocyte growth and maturation can also jeopardize the health and performance of the offspring [33,52,55,56].

In order to replicate the conditions of the maternal reproductive system, techniques used for in vitro embryo production systems must recreate the in vivo environment, with correct regulation of oxygen (O_2_) concentration and composition of culture media [57,58,59]. Several physicochemical factors affect oocytes and embryos, such as temperature control, maintenance of osmolality, pH and protection against oxidative stress and toxic substances [59,60]. De Munck et al. (2019), Leite (2017) and Amin et al. (2014) showed good efficacy of 5% O_2_ during bovine embryo culture [39,58,61]. For cattle oocyte maturation and IVF, the temperature should be 38.8 °C, and it should be performed in atmospheric conditions saturated with humidity and with 5% carbon dioxide (CO_2_) [57,62]. The culture media should recreate the carbohydrate concentrations of the fallopian tubes and uterus, and this can be achieved by adding pyruvate, lactate, glucose and proteins, normally albumin, glutamine and other amino acids [59,63,64]. Antioxidants have been reported also to be necessary [65,66]. Nevertheless, the culture conditions must be adapted to the stage of ART and species, and existing protocols differ between laboratories [6,47,62].

## 3. The Role of Mitochondria in Gamete Functionality

**Figure 2:**
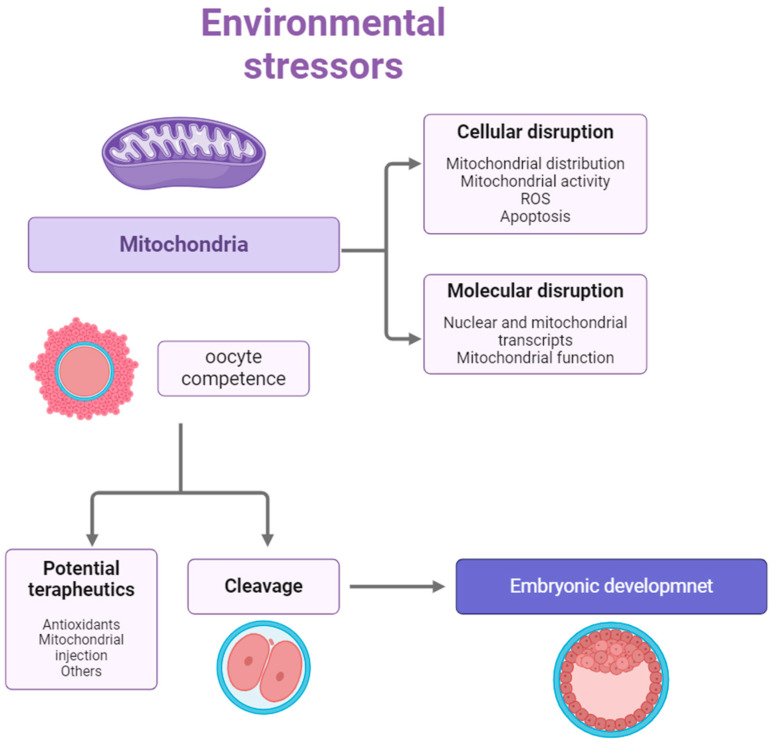
Effects of environmental stress factors on oocyte mitochondria. Stress induces cellular and molecular changes in the oocyte, which in turn can reduce the gamete’s developmental competence. It has been shown that supplementation with mitochondrial agents, antioxidants (epigallocatechin gallate, melatonin) or injection of mitochondria into oocytes attenuates these effects and improves competence for subsequent development (adapted from [8] and created in Biorender.com).

Environmental stressors, including heat stress, significantly affect the developmental competence of gametes. Furthermore, the mitochondrial response to these stressors has been identified as a major cause of reduced oocyte quality (Figure 2) and spermatozoa functionality [8,36,55,65,67]. Mitochondria are cellular organelles, responsible for several metabolic processes, including oxidative phosphorylation (OXPHOS) and fatty acid metabolism, while also regulating cytosolic calcium concentration, the production of ROS and regulating cell death pathways [68,69]. In addition to the nucleus, mitochondria are the only organelles in animal cells that contain their own DNA, known as mitochondrial DNA (mtDNA), which carries essential genetic information [48,70].

Mitochondria use the energy released by the oxidation of glucose and other sugars to synthesize adenosine triphosphate (ATP) from adenosine diphosphate (ADP). Electrons from nicotinamide adenine dinucleotide (NADH) or succinate produced in the mitochondrial matrix during the Krebs cycle are transferred to molecular oxygen by a series of protein complexes in the mitochondrial inner membrane (or in the cristae membranes), creating a transmembrane electrochemical gradient, which is used by the ATP synthase to produce ATP from ADP and inorganic phosphate [71]. This process is called OXPHOS and occurs under aerobic conditions. In addition to energy conversion, this process gives rise to the generation of ROS as by-products of electron transport chain (ETC) activity [72].

Within the oocyte, mitochondria are involved in ATP generation, calcium homeostasis, regulation of cytoplasmic redox status, signal transduction and apoptosis [8]. As oocyte maturation requires a large amount of ATP for continuous transcription and translation, the availability of the right number of functional mitochondria is essential. In addition, during oocyte maturation, the mtDNA copy number increases dramatically and the distribution of mitochondria changes significantly [48,70]. There is therefore a correlation between oocyte quality, mtDNA copy number and ATP concentration [48,70,73].

In spermatozoa, mitochondria are also susceptible to loss of membrane potential and electron leakage during OXPHOS, reducing their energy production capacity [24,74,75,76]. Several studies have analyzed mitochondrial function in sperm cells, establishing a positive correlation between sperm mitochondrial membrane potential (MMP) and other parameters required for sperm functionality, such as motility, viability, capacitation status, acrosome, and chromatin integrity, suggesting that mitochondrial status reflects the quality of the sperm functionality [65,67]. Moreover, according to Jorge et al. (2024), differences among male sperm bioenergetic parameters can be used to predict spermatozoa functionality and developmental potential, and can therefore be used for the selection of bull breeders [64].

On the other hand, if IVF conditions are not optimal, there may be changes in the morphology of mitochondria and in the coding of proteins associated with their function which can lead to a reduced ability to counteract ROS production, leading to oxidative stress [70]. Heat stress is closely associated with alterations in mitochondrial distribution and MMP, disrupting the expression of genes linked to mitochondrial function. This includes genes involved in the transcription and replication of mtDNA and those encoding OXPHOS complexes that are essential for ATP production [8].

Moreover, Soares et al. (2020) have reported significant age-related alterations in bovine oocytes, namely in the cytoplasmic volume, mitochondrial aggregation pattern and mitochondria-produced H_2_O_2_ levels [77]. Aged bovine oocytes have also presented reduced nuclear maturation progression, MMP, developmental competence and altered gene expression levels [55]. Those unique characteristics mean that germplasm mitochondria can be used to study fertility/infertility and to test new therapeutic strategies that aim to improve the mitochondrial function of gametes and embryos.

## 4. Reactive Oxygen Species (ROS) in Gametes and Embryos

**Figure 3:**
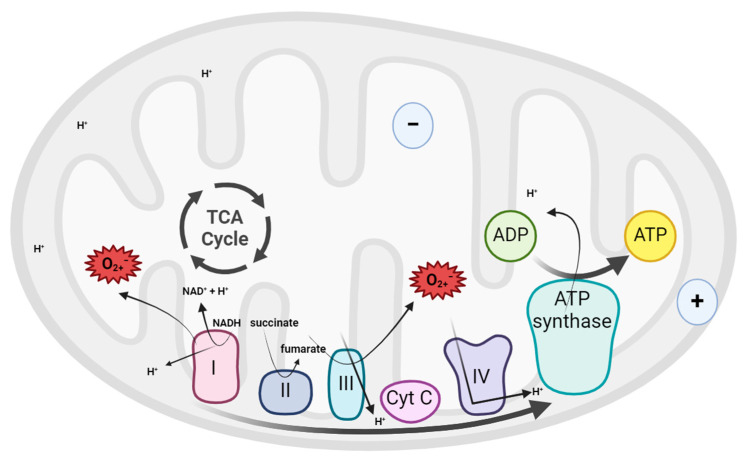
Respiratory chain in mitochondria. During aerobic respiration, electrons from NADH and succinate are transferred to molecular O_2_ through a chain of redox enzymes (complexes I–IV) embedded in the inner mitochondrial membrane, which is accompanied by pump protons across the inner membrane, generating an electrochemical gradient. This proton gradient is used for the mechanical work needed to produce ATP from ADP and inorganic phosphate (Created in Biorender.com).

Oxidation-reduction reactions are a basic component of the systems of living beings and the free radicals, including oxygen ions and hydrogen peroxide, that result from them, are also essential for nuclear maturation or sperm capacitation. These radicals are the result of cellular metabolism and enzymatic reactions that occur in the body and require a balance between the loss and gain of electrons [8,65,66,78,79]. As previously stated, during OXPHOS, a residual production of ROS occurs naturally, as a consequence of electron transfer. Studies focusing on specific complexes embedded in the mitochondrial inner membrane have established that complexes I and III are the sites with the greatest capacity for generating ROS, due to electron slippage [72,80] (Figure 3).

ROS play direct and indirect roles in a very wide range of physiological processes. However, when there is an imbalance in their homeostasis, i.e., an imbalance between oxidants and antioxidants in favor of the oxidants, this leads to a disruption of redox signaling and control and/or molecular damage [65,66,78,79,81]. Oxidative stress and mitochondrial dysfunction have been associated with metabolic diseases and age.

The presence of excess ROS within the ovary and endometrium has significant physiological and pathological implications for conception [79]. In IVF, there are various sources of ROS, including the cells themselves, such as oocytes and spermatozoa, as well as the composition of the culture media used. In addition, the laboratory culture conditions used during maturation and insemination, namely excess light, temperature variation, and high oxygen tension, increase the production of ROS and can have detrimental effects on post-fertilization development and assisted reproduction results, as they have a detrimental effect on mitochondria, DNA, RNA and fertilization [36,38,66].

An increase in ROS is well documented in oocytes exposed to heat stress or environmental toxicants [8,23] and, in spermatozoa, this causes damage to membrane lipids and decreased sperm motility. The exposure of DNA to both ROS and apoptosis enzymes during the cell division of spermatogenesis makes spermatozoa vulnerable, impairing not only fertilization but also subsequent embryonic development [24,75,82]. In addition, this increase in ROS production and consequent oxidative stress affects the quality of gametes, their environment, and ultimately, their interaction, which can not only reduce IVF success rates but can also result in epigenetic and genetic changes in the embryo, resulting in transgenerational effects [36,39,61,66].

Marques et al. [83] detected the production of mitochondria-specific ROS (mtROS) in sperm using flow cytometry with a mitochondria-specific superoxide fluorescent probe (MitoSOX Red). In this study, they showed that ejaculates were heterogeneous in mtROS production, with three detectable subpopulations, and the sperm subpopulation that produced the least amount of mtROS presented the most functional subset of male gametes, which were correlated with the largest number of live gametes and non-apoptotic sperm. In addition, this subpopulation was clearly more effective in samples that gave rise to pregnancies after assisted reproduction. Recently, Nakai et al. [84] have shown that increased ROS production was associated with oocyte maturation inhibition and demonstrated that the loss of a protein membrane (Mul1) was directly connected with the increase of ROS concentrations in oocytes, resulting in an abnormal preimplantation embryogenesis. The results of this study emphasized that manipulating the mitochondrial ROS levels in oocytes may be a potential therapeutic approach to target infertility [84]

Several studies have therefore concluded that the use of antioxidants is an effective approach to mitigate oxidative stress and improve fertility [36,85,86]. Agarwal et al. [36] stated that infertility treatment strategies should focus on oral supplementation with antioxidants or, in the case of IVF, supplementation of the culture media with antioxidants to reduce oxidative stress.

## 5. Antioxidants in Reproductive Medicine

Antioxidants are substances that inhibit oxidation by neutralizing free radicals found in the environment, reducing their effects on various diseases such as cancer, diabetes, degenerative diseases, aging and infertility [87]. Eukaryotic cells have antioxidant strategies and multifaceted mechanisms to maximize energy production, while limiting the negative and toxic effects of ROS production [72]. There are several natural antioxidants in the body, including enzymatic antioxidants such as catalase, SOD and glutathione peroxidase [79,88]. In addition, non-enzymatic antioxidants such as glutathione (GSH), vitamins C and E, hypotaurine and taurine, among others, provide maternal protection from oxidative stress to oocytes and embryos [45].

Excessive ROS production, if not counteracted by intrinsic defense mechanisms, will cause oxidative damage to cellular components and, as mentioned above, can lead to DNA damage and cell death by necrosis or apoptosis [8,78,89,90,91]. Thus, antioxidant supplementation can be a strategy to improve fertility. Moreover, the use of antioxidants has been reported to be necessary to enhance ART success [65,66], as summarized in Table 1 and described below.

### 5.1. Endogenous Antioxidants

**Figure 4:**
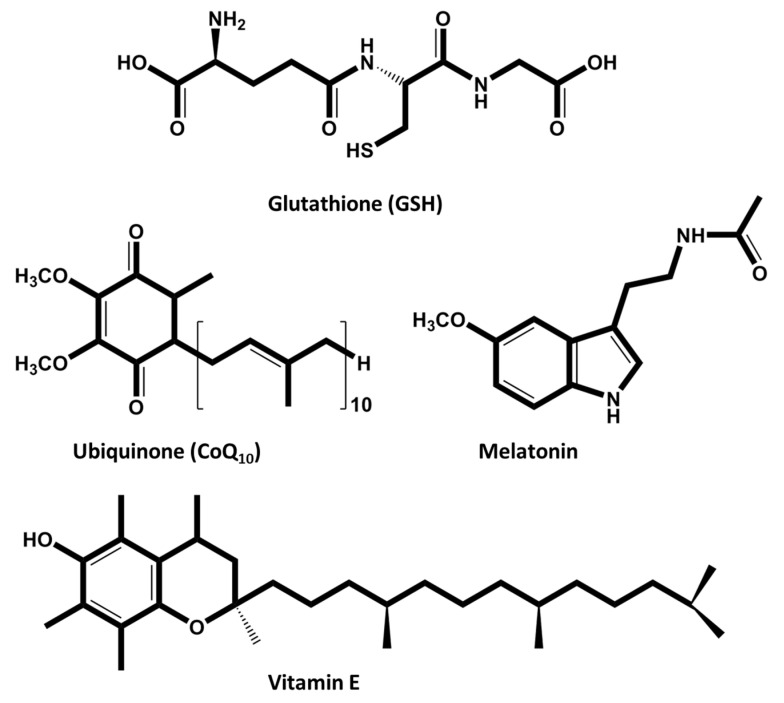
Chemical structures of endogenous antioxidants evaluated in an ART context. Glutathione (GSH), ubiquinone (CoQ_10_), melatonin, and vitamin E have been tested in bovine germplasm to improve fertility.

Reduced glutathione (GSH) is an endogenous tripeptide antioxidant (Figure 4) present in both male and female gametes that plays a very important role in fertility. GSH has a role in intracellular defense against oxidative stress and contributes to the regeneration of other antioxidants. [92,93]. A study by Ogata et al. [92] has shown positive effects on the early formation of male pronuclei without negative effects on DNA integrity and cell number in blastocyst stage embryos. Although the effect was dependent on the bull and GSH concentration, semen supplementation improved in vitro embryo production from frozen semen. In this study, after GSH supplementation (0, 1, 5 and 10 mM), sperm quality varied between bulls. One bull had decreased total sperm motility and two other bulls had decreased sperm DNA integrity. GSH supplementation had positive effects on embryonic development in all three bulls. Two of them showed improved cleavage rates and blastocyst formation, while the other only showed a higher cleavage rate. In addition to GSH, to counteract the harmful effects of ROS, many studies have included vitamin E in semen extenders or embryo culture media to improve cryosurvival parameters, sperm cell viability and embryo production [94,95,96]. Vitamin E is a well-established, naturally-occurring, lipid-soluble, chain-breaking antioxidant that scavenges oxygen radicals within the membranes, acting in synergy with vitamin C and protecting germlines [96,106].

Coenzyme Q10 (CoQ10), or ubiquinone (Figure 4), which is a free radical scavenger and a very important component of mitochondrial ETC has also been found to impact male and female gametes [8,23,114]. CoQ10’s antioxidant function (ubiquinol) works by inhibiting lipid peroxidation in vitro and in vivo. In addition to inhibiting lipid peroxidation without the mediation of vitamin E, ubiquinol can also amplify the antioxidant effect of this vitamin. It should be noted that ubiquinol is the only known fat-soluble antioxidant that can be synthesized in animal cells and regenerated through the mitochondrial electron transport system to its reduced antioxidant form. These characteristics, together with its high degree of hydrophobicity and its general occurrence in biological membranes and low-density lipoproteins, suggest a very important role for ubiquinol in cellular defense against oxidative damage [115,116].

Gendelman & Roth (2012) found that an oocyte maturation medium supplemented with 50 μM of CoQ10 induced changes in mitochondrial distribution within the oocytes and increased the proportion of polarized mitochondria. In addition, CoQ10 incorporation into oocytes induced changes in gene transcription that were involved in the mitochondrial ETC, and led to a higher proportion of embryos that developed into blastocysts. However, CoQ10 did not improve oocyte development in winter and summer, which suggests that this antioxidant only contributes beneficially in phases with moderate climate damage, as there was an improvement in the fall [23]. Even so, these authors have suggested the widespread use of this antioxidant to increase the in vitro production of bovine embryos.

In 2017, Yang et al. tested the action of the natural antioxidant melatonin (10^−9^ M) (Figure 4) in IVF, demonstrating positive results in the maturation of bovine oocytes and an increase in the number of produced embryos; they concluded that melatonin improves the distribution of mitochondria and preserves ATP production [45].

### 5.2. Exogenous Antioxidants

Natural antioxidants derived from the diet have antioxidative characteristics, with the potential to prevent diseases caused by oxidative stress [87]. Antioxidants in fruits, vegetables and drinks play an important role in mammalian health, such as preventing cancer and cardiovascular disease and reducing the incidence of different diseases [126]. The study of these natural compounds in food/feed will contribute to increasing their use, rather than the use of artificial drugs.

#### 5.2.1. Naturally-Occurring Antioxidants

**Figure 5:**
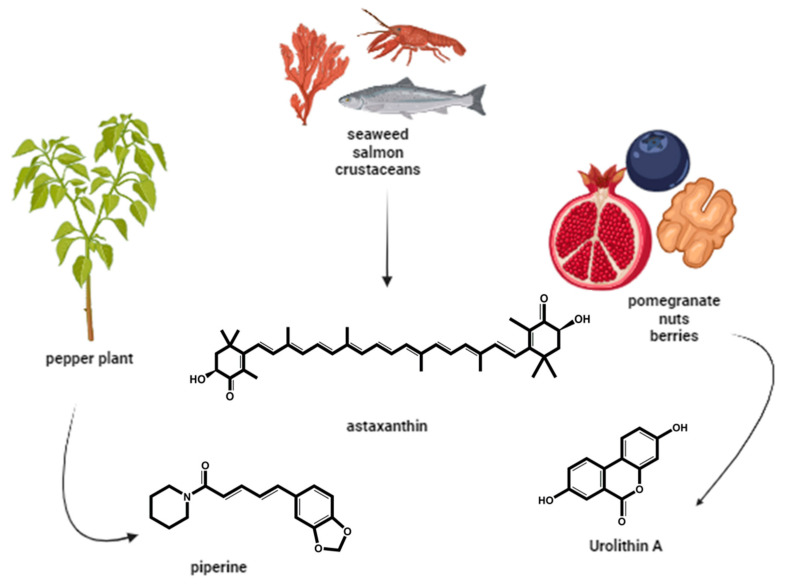
Exogenous naturally occurring antioxidants used to improve ART outcomes. Piperine, astaxanthin and urolithin A have been tested in animal germplasm to improve fertility (created in BioRender.com).

Piperine is a simple alkaloid found in black pepper seeds (Figure 5), whose biological properties have been extensively studied at the pharmacological level and it has shown great therapeutic potential as an antioxidant, anticancer, anti-inflammatory, antihypertensive, hepatoprotective and neuroprotective agent, as well as in improving fertility. It also can alter gastrointestinal disorders and drug metabolizing enzymes [97,98].

Piperine induces specific toxicity in cancer cells, but does not affect normal cells, and has also been shown to restore the function of aged cells around cancer cells [99]. In addition, piperine improves the efficacy of current cancer therapies and represents a good adjuvant to certain phytochemical compounds, such as curcumin or resveratrol.

The use of piperine as a bioactive compound is still in its infancy, so studies on this compound are still essential for the formulation, improvement and discovery of new drugs. Recently, our team has used piperine during bovine oocyte maturation. It was demonstrated that 1 and 10 µM piperine improved the number of mature oocytes (metaphase II.), and higher MMP was attained with 1 µM concentration compared to the control [100].

Polyphenols are secondary plant metabolites that are mostly involved in defense against oxidative stress and are found largely in fruits, vegetables, cereals and beverages [102,123,124]. Over the last few years, several studies have found that foods rich in polyphenols protect against age-related diseases, such as atherosclerosis, cardiovascular disease, cancer, arthritis, cataracts, osteoporosis, type 2 diabetes mellitus, hypertension, Alzheimer’s disease and diseases with mitochondrial etiology [102,125].

In 2024, Yang et al. showed a positive effect of supplementation with the natural antioxidant astaxanthin (Figure 5) on porcine granulosa cells’ culture, identifying positive variations in morphology, apoptosis, ROS levels and the expression of apoptosis and anti-oxidation-related genes [107]. Astaxanthin has also been found to ameliorate oxidative stress and reproductive outcomes after assisted reproduction [108].

Another example of an antioxidant of natural origin is urolithin A (Figure 5), which is a metabolite resulting from the transformation by intestinal bacteria of ellagitannins, ellagic acid and polyphenols, found in foods such as pomegranates, berries and nuts. Several studies have shown that it plays an important role in preventing aging and various diseases [55,117,118]. Fonseca et al. [55] tested the use of this antioxidant in the development of oocytes from old and young cows and concluded that supplementing the oocyte maturation medium with this antioxidant prevented oocyte aging and improved embryonic development. Later, Jorge et al. [64] reinforced the potential therapeutic value of urolithin A for addressing reproductive sub/infertility problems and improving ART outcomes. Their results showed that urolithin A improved sperm motility quality, increasing ATP production while reducing oxidative stress levels in a dose-dependent manner.

#### 5.2.2. Synthetic Mitochondriotropic Antioxidants

For several years, different approaches have been established to prevent oxidative damage to mitochondria, including the development of ETC inhibitors, OXPHOS and mitochondrial Ca^2+^ modulators and mitochondriotropic antioxidants [90]. The pharmacological activities of new naturally derived and synthetic molecules have been extensively studied [87]. Currently, the prevention of mitochondrial oxidative damage through pharmacological solutions is recognized as an indispensable tool [55,102] to strengthen mitochondria’s antioxidant power. Mitochondrial function can be improved through the use of antioxidants, and various types of antioxidants have already been tested in several animal species and human oocytes with promising results [70], especially when targeted at the mitochondria [64,65,121].

**Figure 6:**
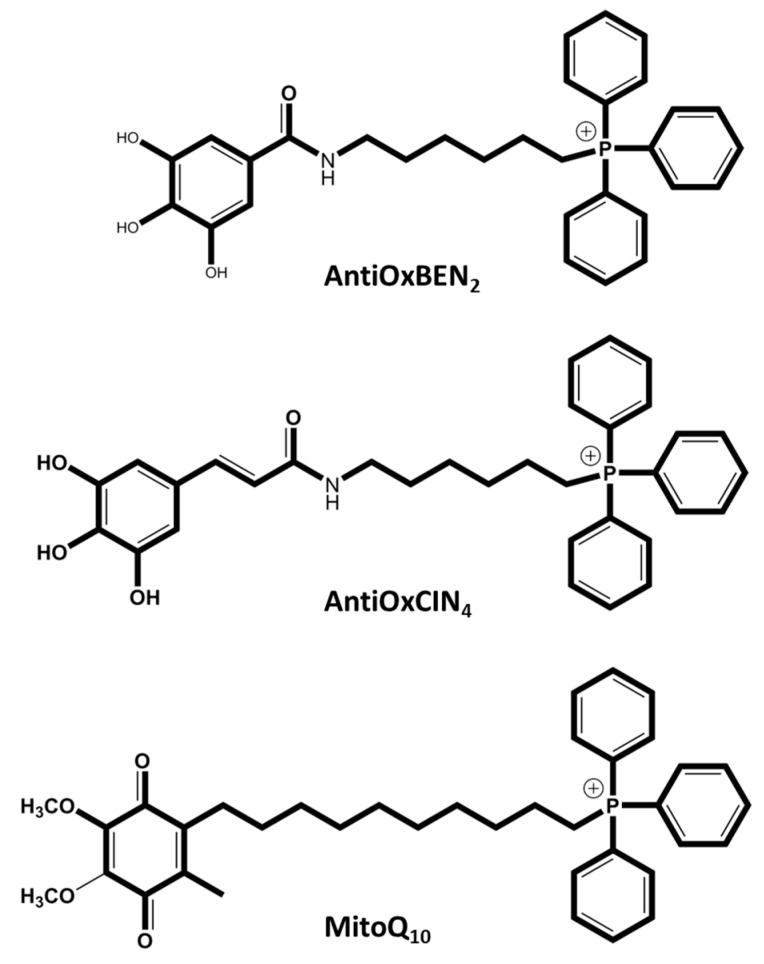
Chemical structures of synthetic mitochondriotropic antioxidants evaluated in an ART context. AntiOxBEN_2_, AntiOxCIN_4_, and MitoQ_10_ have been tested in bovine cells to decreased ROS and increase the results of ART.

Mitoquinone (MitoQ) is one of the most widely studied mitochondriotropic antioxidants. MitoQ has shown encouraging pre-clinical results in numerous studies on isolated mitochondria, cells and tissues subjected to oxidative stress and death by apoptosis, as it can reproduce the role of the endogenous mitochondrial antioxidant CoQ10. This coenzyme is covalently linked to a triphenylphosphonium cation (TPP) by a 10-carbon alkyl chain (dTPP), a lipophilic spacer (Figure 6) that allows the molecule to cross mitochondrial membranes and considerably increases its antioxidant capacity. This antioxidant represents the first clinical attempt to deliver an antioxidant to the mitochondria. The results of clinical trials with MitoQ were very important for understanding the relevance of a mitochondria-oriented approach [101,102].

A study in bovine oocytes demonstrated that a maturation culture medium supplemented with MitoQ (1 µM) improved mitochondrial function and enhanced embryo development. This effect was achieved by reducing mitochondrial ROS levels below the critical thresholds that trigger apoptosis [103]. Another study carried out during the maturation of rat oocytes also concluded that the addition of MitoQ (0, 0.01, 0.02 and 0.04 µM) to IVF culture media improved oocyte fertilization and the subsequent development of blastocysts [127]. An increased GSH level and membrane potential of oocytes concomitant with reduced intracellular ROS concentrations were identified. In addition, supplementation of a stallion semen extender with MitoQ (25, 50, and 100 nM) before cryopreservation showed a positive effect of this antioxidant on sperm mobility, especially at 25 nM. The highest concentration of 200 nM harmed the mobility and viability parameters of frozen–thawed semen. Nevertheless, none of the concentrations used affected the plasma membrane, acrosome, DNA integrity, MMP or intracellular ROS concentrations [104]. Conversely, the addition of MitoQ (0, 0.2, 2 and 20 nM) to the semen extender before cryopreservation was unable to improve the post-thaw quality of bull sperm [128]. According to some studies, MitoQ supplementation harmed the bioenergetic function of mitochondria and did not contribute to the inhibition of iron toxicity [102,105].

AntiOxBEN_2_ is a mitochondria-targeted antioxidant derived from gallic acid, which is a hydroxybenzoic acid. AntiOxBEN_2_ has been synthesized by conjugating the antioxidant gallic acid with the lipophilic triphenylphosphonium cation (TPP+) (Figure 6) through a 6-carbon aliphatic chain, designed to specifically accumulate within the mitochondrial matrix [102,109,110]. Results of in vitro models show that AntiOxBEN2, unlike MitoQ10, proved capable of chelating ferrous iron, upregulating antioxidant systems and improving mitochondria function by activating the Nrf2/Keap1 pathway [111]. Teixeira et al. [121] studied the effect of the mitochondriotropic antioxidant AntiOxBEN_2_ on the prevention of oxidative stress in bovine oocytes and embryos by supplementing the oocyte maturation medium with concentrations of 10, 20, 50 and 100 μM. AntiOxBEN_2_ improved oocyte maturation and embryo production in a dose-dependent manner. These authors suggested its future use at a concentration of 10 μM during the oocyte maturation process. Later, Santos et al. [65] supplemented culture media during IVF of bovine oocytes with AntiOxBEN_2_, demonstrating its benefits in reducing ROS, increasing spermatozoa MMP and consequently sperm quality. When the sperm capacitation and fertilization media were supplemented with a concentration of 1 µM of AntiOxBEN_2_, an improvement in embryo development, due to an increase of the number of cleaved embryos and blastocysts, was seen.

AntiOxCIN_4_ is a mitochondriotropic antioxidant based on caffeic acid that has been observed to prevent oxidative stress-related events through the activation of endogenous ROS-protective pathways in normal primary human fibroblasts (PHSF) and in PHSF from sporadic Parkinson’s disease patients (Figure 6). AntiOxCIN_4_ also increased cell stress resistance in human hepatoma-derived cells (HepG2) by activating the Nrf2-p62-Keap1 axis, leading to up-regulation of antioxidant defenses, triggering macroautophagy and/or mitochondrial autophagy (mitophagy) and mitochondrial biogenesis [105,119]. Additionally, AntiOxCIN_4_ supplementation has been shown to improve steatotic liver disease in a metabolic disease mice model [120]. Teixeira et al. (2020) supplemented the bovine oocyte maturation medium with concentrations of 10, 20, 50 and 100 μM of AntiOxCIN_4_, which was shown to improve oocyte maturation in a dose-dependent manner [121]. Recently, supplementation of the capacitation and fertilization media with AntiOxCIN_4_ at concentrations of 0.1 and 1 μM has been studied. It was found that supplementation with 1 μM AntiOxCIN_4_ during the bull sperm capacitation process improved some of the functional characteristics of the spermatozoa and that both concentrations (0.1 and 1 μM) increased the number of good quality embryos. However, the study is still ongoing, in order to produce more robust data [122].

Finally, AntiOxBEN_2_ and AntiOxCIN_4_ antioxidants at concentrations of 1, 2.5 and 10 μM have also been tested in bovine embryo culture media to prevent oxidative stress. The concentration of 2.5 μM improved the quality of the produced embryos. Based on these results, this concentration was used to further study the resistance of these embryos to the vitrification process. The results showed that AntiOxCIN_4_ and especially AntiOxBEN_2_ had a beneficial effect on embryo development and cryopreservation survival, pointing to a possible therapy to prevent oxidative stress in ART [112,113].

## 6. Conclusions

This review has shown that fertility problems and ART use are increasing worldwide in several species. The reasons given range from human-induced environmental changes, with its repercussions on pollution and all its surroundings, to climate change and production demands. At present, ART procedures are imperative to overcome fertility problems and to maintain animal productivity and biodiversity. The study of natural antioxidants, especially those targeting mitochondria, is essential, because mitochondria play a crucial role in the generation of ROS and oxidative stress, which contribute to low fertility rates. These compounds have been shown to have multiple and beneficial roles, with promising results in bovine ART. However, further research including other species is needed to broaden the existing data.

## Figures and Tables

**Table 1:** Brief summary of the different antioxidants under investigation in various studies to enhance ART outcomes.

Antioxidants					
Endogenous Antioxidants		Naturally-Occurring Exogenous Antioxidants		Synthetic Mitochondriotropic Antioxidants	
Reduced glutathione (GSH)	[45,92,93,94,95,96]	Piperine	[97,98,99,100]	Mitoquinone (MitoQ)	[101,102,103,104,105]
Vitamin E and C	[45,96,106]	Astaxanthin	[107,108]	AntiOxBEN_2_	[65,102,109,110,111,112,113]
Coenzyme Q10	[8,23,114,115,116]	Urolithin A	[55,64,117,118]	AntiOxCIN_4_	[105,112,113,119,120,121,122]
Melatonin	[45]	Polyphenols	[102,123,124,125]		
Hypotaurine and taurine	[45]				

## Data Availability

Not applicable.

## References

[1] Luoma J. Challenged Conceptions: Environmental Chemicals and Fertility. Proceedings of the Understanding Environmental Contaminants and Human Fertility: Science and Strategy.

[2] Canipari R., De Santis L., Cecconi S. (2020). Female Fertility and Environmental Pollution. Int. J. Environ. Res. Public. Health.

[3] Yin D., Mao R., Wang D., Yu P., Zhou C., Liu J., Li S., Nie Y., Liao H., Peng C. (2024). Association of Plasma Metal Levels with Outcomes of Assisted Reproduction in Polycystic Ovary Syndrome. Biol. Trace Elem. Res..

[4] Shen J., Mao Y., Zhang H., Lou H., Zhang L., Moreira J.P., Jin F. (2024). Exposure of Women Undergoing In-Vitro Fertilization to per-and Polyfluoroalkyl Substances: Evidence on Negative Effects on Fertilization and High-Quality Embryos. Environ. Pollut..

[5] Gallo A., Boni R., Tosti E. (2020). Gamete Quality in a Multistressor Environment. Environ. Int..

[6] Pereira R., Marques C.C., Pimenta J., Barbas J.P., Baptista M.C., Diniz P., Torres A., Lopes-da-Costa L. (2020). Assisted Reproductive Technologies (ART) Directed to Germplasm Preservation. Advances in Animal Health, Medicine and Production.

[7] Sakali A.-K., Bargiota A., Bjekic-Macut J., Macut D., Mastorakos G., Papagianni M. (2024). Environmental Factors Affecting Female Fertility. Endocrine.

[8] Roth Z. (2018). Symposium Review: Reduction in Oocyte Developmental Competence by Stress Is Associated with Alterations in Mitochondrial Function1. J. Dairy Sci..

[9] LaPointe S., Lee J.C., Nagy Z.P., Shapiro D.B., Chang H.H., Wang Y., Russell A.G., Hipp H.S., Gaskins A.J. (2024). Air Pollution Exposure in Vitrified Oocyte Donors and Male Recipient Partners in Relation to Fertilization and Embryo Quality. Environ. Int..

[10] Santos R.R., Schoevers E.J., Roelen B.A.J. (2014). Usefulness of Bovine and Porcine IVM/IVF Models for Reproductive Toxicology. Reprod. Biol. Endocrinol..

[11] Sharara F.I., Seifer D.B., Flaws J.A. (1998). Environmental Toxicants and Female Reproduction. Fertil. Steril..

[12] Mattison D.R. (1983). The Mechanisms of Action of Reproductive Toxins. Am. J. Ind. Med..

[13] Faber R.A., Hickey J.J. (1973). Eggshell Thinning, Chlorinated Hydrocarbons, and Mercury in Inland Aquatic Bird Eggs, 1969 and 1970. Pestic Monit. J..

[14] Leroy J.L.M.R., Van Soom A., Opsomer G., Goovaerts I.G.F., Bols P.E.J. (2008). Reduced Fertility in High-Yielding Dairy Cows: Are the Oocyte and Embryo in Danger? Part II. Mechanisms Linking Nutrition and Reduced Oocyte and Embryo Quality in High-Yielding Dairy Cows. Reprod. Domest. Anim..

[15] Gameiro S. (2016). Infertility. Encyclopedia of Mental Health.

[16] Kiesswetter M., Marsoner H., Luehwink A., Fistarol M., Mahlknecht A., Duschek S. (2020). Impairments in Life Satisfaction in Infertility: Associations with Perceived Stress, Affectivity, Partnership Quality, Social Support and the Desire to Have a Child. Behav. Med..

[17] Szkodziak P., Wozniak S., Czuczwar P., Wozniakowska E., Milart P., Mroczkowski A., Paszkowski T. (2016). Infertility in the Light of New Scientific Reports–Focus on Male Factor. Ann. Agric. Environ. Med..

[18] Dominguez A.A., Reijo Pera R.A. (2013). Infertility. Brenner’s Encyclopedia of Genetics.

[19] Gaskins A.J., Chavarro J.E. (2018). Diet and Fertility: A Review. Am. J. Obstet. Gynecol..

[20] Zhang M., Peng W.F., Hu X.J., Zhao Y.X., Lv F.H., Yang J. (2018). Global Genomic Diversity and Conservation Priorities for Domestic Animals Are Associated with the Economies of Their Regions of Origin. Sci. Rep..

[21] Yawer A., Sychrová E., Labohá P., Raška J., Jambor T., Babica P., Sovadinová I. (2020). Endocrine-Disrupting Chemicals Rapidly Affect Intercellular Signaling in Leydig Cells. Toxicol. Appl. Pharmacol..

[22] Guillette L.J., Edwards T.M. (2005). Is Nitrate an Ecologically Relevant Endocrine Disruptor in Vertebrates?. Integr. Comp. Biol..

[23] Gendelman M., Roth Z. (2012). Incorporation of Coenzyme Q10 into Bovine Oocytes Improves Mitochondrial Features and Alleviates the Effects of Summer Thermal Stress on Developmental Competence. Biol. Reprod..

[24] Capela L., Leites I., Romão R., Lopes-Da-costa L., Pereira R.M.L.N. (2022). Impact of Heat Stress on Bovine Sperm Quality and Competence. Animals.

[25] Nandi S., Tripathi S.K., Gupta P.S.P., Mondal S. (2018). Nutritional and Metabolic Stressors on Ovine Oocyte Development and Granulosa Cell Functions in Vitro. Cell Stress Chaperones.

[26] Hm Viana J. (2021). 2020 Statistics of Embryo Production and Transfer in Domestic Farm Animals. Embryo Technol. Newsl..

[27] Galli C., Lazzari G. (1996). Practical Aspects of IVM/IVF in Cattle. Anim. Reprod. Sci..

[28] Parker Gaddis K.L., Dikmen S., Null D.J., Cole J.B., Hansen P.J. (2017). Evaluation of Genetic Components in Traits Related to Superovulation, in Vitro Fertilization, and Embryo Transfer in Holstein Cattle. J. Dairy Sci..

[29] Yaginuma H., Funshima N., Tanikawa N., Miyamura M., Tsuchiya H., Noguchi T., Iwata H., Kuwayama T., Shirasuna K., HAMANO S. (2019). Improvement of Fertility in Repeat Breeder Dairy Cattle by Embryo Transfer Following Artificial Insemination: Possibility of Interferon Tau Replenishment Effect. J. Reprod. Dev..

[30] Funeshima N., Noguchi T., Onizawa Y., Yaginuma H., Mitamura M., Tsuchiya H., Iwata H., Kuwayama T., Hamano S., Shirasuna K. (2019). The Transfer of Parthenogenetic Embryos Following Artificial Insemination in Cows Can Enhance Pregnancy Recognition via the Secretion of Interferon Tau. J. Reprod. Dev..

[31] Hansen P.J. (2006). Realizing the Promise of IVF in Cattle—An Overview. Theriogenology.

[32] Siqueira L.G.B., Dikmen S., Ortega M.S., Hansen P.J. (2017). Postnatal Phenotype of Dairy Cows Is Altered by in Vitro Embryo Production Using Reverse X-Sorted Semen. J. Dairy Sci..

[33] Leroy J.L.M.R., Rizos D., Sturmey R., Bossaert P., Gutierrez-Adan A., Van Hoeck V., Valckx S., Bols P.E.J. (2012). Intrafollicular Conditions as a Major Link between Maternal Metabolism and Oocyte Quality: A Focus on Dairy Cow Fertility. Reprod. Fertil. Dev..

[34] Malhi P.S., Adams G.P., Mapletoft R.J., Singh J. (2007). Oocyte Developmental Competence in a Bovine Model of Reproductive Aging. Reproduction.

[35] Moore S.G., Hasler J.F. (2017). A 100-Year Review: Reproductive Technologies in Dairy Science. J. Dairy Sci..

[36] Agarwal A., Gupta S., Sharma R. (2005). Oxidative Stress and Its Implications in Female Infertility-A Clinician’s Perspective. Reprod. Biomed. Online.

[37] Song P., Liu C., Sun M., Liu J., Lin P., Wang A., Jin Y. (2022). Oxidative Stress Induces Bovine Endometrial Epithelial Cell Damage through Mitochondria-Dependent Pathways. Animals.

[38] Agarwal A., Said T.M., Bedaiwy M.A., Banerjee J., Alvarez J.G. (2006). Oxidative Stress in an Assisted Reproductive Techniques Setting. Fertil. Steril..

[39] Leite R.F., Annes K., Ispada J., de Lima C.B., dos Santos É.C., Fontes P.K., Gouveia Nogueira M.F., Milazzotto M.P. (2017). Oxidative Stress Alters the Profile of Transcription Factors Related to Early Development on in Vitro Produced Embryos. Oxidative Med. Cell. Longev..

[40] Pereira R., Marques C. (2017). Produção e Transferência de Embriões: Uma Técnica Em Expansão; Embryo production and transfer: A technique in expansion. Vida Rural.

[41] Verberckmoes S., Van Soom A., De Kruif A. (2004). Intra-Uterine Insemination in Farm Animals and Humans. Reprod. Domest. Anim..

[42] Morris L.H.A. (2018). The Development of in Vitro Embryo Production in the Horse. Equine Vet. J..

[43] Hinrichs K. (2018). Assisted Reproductive Techniques in Mares. Reprod. Domest. Anim..

[44] Inhorn M.C., Patrizio P. (2014). Infertility around the Globe: New Thinking on Gender, Reproductive Technologies and Global Movements in the 21st Century. Hum. Reprod. Update.

[45] Yang M., Tao J., Chai M., Wu H., Wang J., Li G., He C., Xie L., Ji P., Dai Y. (2017). Melatonin Improves the Quality of Inferior Bovine Oocytes and Promoted Their Subsequent IVF Embryo Development: Mechanisms and Results. Molecules.

[46] Rizos D., Clemente M., Bermejo-Alvarez P., de La Fuente J., Lonergan P., Gutiérrez-Adán A. (2008). Consequences of *In Vitro* Culture Conditions on Embryo Development and Quality. Reprod. Domest. Anim..

[47] Pereira R.M., Marques C.C. (2008). Animal Oocyte and Embryo Cryopreservation. Cell Tissue Bank..

[48] Kageyama M., Ito J., Shirasuna K., Kuwayama T., Iwata H. (2021). Mitochondrial Reactive Oxygen Species Regulate Mitochondrial Biogenesis in Porcine Embryos. J. Reprod. Dev..

[49] Palermo G.D., Neri Q.V., Rosenwaks Z. (2015). To ICSI or Not to ICSI. Semin. Reprod. Med..

[50] Carnevale E.M., Maclellan L.J., Stokes J.A.E. (2019). In Vitro Culture of Embryos from Horses. Methods in Molecular Biology.

[51] Bedoschi G., Roque M., Esteves S.C. (2020). ICSI and Male Infertility: Consequences to Offspring. Male Infertility.

[52] Duranthon V., Chavatte-Palmer P. (2018). Long Term Effects of ART: What Do Animals Tell Us?. Mol. Reprod. Dev..

[53] Pontes J.H.F., Nonato-Junior I., Sanches B.V., Ereno-Junior J.C., Uvo S., Barreiros T.R.R., Oliveira J.A., Hasler J.F., Seneda M.M. (2009). Comparison of Embryo Yield and Pregnancy Rate between in Vivo and in Vitro Methods in the Same Nelore (Bos Indicus) Donor Cows. Theriogenology.

[54] Rasmussen S., Block J., Seidel G.E., Brink Z., McSweeney K., Farin P.W., Bonilla L., Hansen P.J. (2013). Pregnancy Rates of Lactating Cows after Transfer of in Vitro Produced Embryos Using X-Sorted Sperm. Theriogenology.

[55] Fonseca É., Marques C.C., Pimenta J., Jorge J., Baptista M.C., Gonçalves A.C., Pereira R.M.L.N. (2021). Anti-Aging Effect of Urolithin a on Bovine Oocytes in Vitro. Animals.

[56] Igarashi H., Takahashi T., Takahashi E., Tezuka N., Nakahara K., Takahashi K., Kurachi H. (2005). Aged Mouse Oocytes Fail to Readjust Intracellular Adenosine Triphosphates at Fertilization. Biol. Reprod..

[57] Pereira R.M., Marques C.C., Baptista M.C., Vasques M.I., Horta A.E.M. (2009). Embryos and Culture Cells: A Model for Studying the Effect of Progesterone. Anim. Reprod. Sci..

[58] De Munck N., Janssens R., Segers I., Tournaye H., Van De Velde H., Verheyen G. (2019). Influence of Ultra-Low Oxygen (2%) Tension on in-Vitro Human Embryo Development. Hum. Reprod..

[59] Cohen J., Rieger D., Mastenbroek S., Meintjes M., Janssens R., Catt J., Morbeck D., Mortimer D., Fawzy M., Alikani M. (2020). ‘There Is Only One Thing that Is Truly Important in an IVF Laboratory: Everything’ Cairo Consensus Guidelines on IVF Culture Conditions. Reprod. Biomed. Online.

[60] Mortimer D., Cohen J., Mortimer S.T., Fawzy M., McCulloh D.H., Morbeck D.E., Pollet-Villard X., Mansour R.T., Brison D.R., Doshi A. (2018). Cairo Consensus on the IVF Laboratory Environment and Air Quality: Report of an Expert Meeting. Reprod. BioMedicine Online.

[61] Amin A., Gad A., Salilew-Wondim D., Prastowo S., Held E., Hoelker M., Rings F., Tholen E., Neuhoff C., Looft C. (2014). Bovine Embryo Survival under Oxidative-Stress Conditions Is Associated with Activity of the NRF2-Mediated Oxidative-Stress-Response Pathway. Mol. Reprod. Dev..

[62] Marques C.C., Santos-Silva C., Rodrigues C., Matos J.E., Moura T., Baptista M.C., Horta A.E.M., Bessa R.J.B., Alves S.P., Soveral G. (2018). Bovine Oocyte Membrane Permeability and Cryosurvival: Effects of Different Cryoprotectants and Calcium in the Vitrification Media. Cryobiology.

[63] Tay J.I., Rutherford\ A.J., Killick S.R., Maguiness S.D., Partridge R.J., Leese H.J. (1997). Human Tubal Fluid: Production, Nutrient Composition and Response to Adrenergic Agents. Hum. Reprod..

[64] Jorge M., Ferreira F.C., Marques C.C., Batista M.C., Oliveira P.J., Lidon F., Duarte S.C., Teixeira J., Pereira R.M.L.N. (2024). Effect of Urolithin A on Bovine Sperm Capacitation and In Vitro Fertilization. Animals.

[65] Santos J.C., Marques C.C., Baptista M.C., Pimenta J., Teixeira J., Montezinho L., Cagide F., Borges F., Oliveira P.J., Pereira R.M.L.N. (2022). Effect of a Novel Hydroxybenzoic Acid Based Mitochondria Directed Antioxidant Molecule on Bovine Sperm Function and Embryo Production. Animals.

[66] Hardy M.L.M., Day M.L., Morris M.B. (2021). Redox Regulation and Oxidative Stress in Mammalian Oocytes and Embryos Developed in Vivo and in Vitro. Int. J. Environ. Res. Public. Health.

[67] Sousa A.P., Amaral A., Baptista M., Tavares R., Campo P.C., Peregrín P.C., Freitas A., Paiva A., Almeida-Santos T., Ramalho-Santos J. (2011). Not All Sperm Are Equal: Functional Mitochondria Characterize a Subpopulation of Human Sperm with Better Fertilization Potential. PLoS ONE.

[68] Agrawal A., Mabalirajan U. (2016). Rejuvenating Cellular Respiration for Optimizing Respiratory Function: Targeting Mitochondria. Am. J. Physiol. Lung Cell Mol. Physiol..

[69] Wallace D.C., Fan W., Procaccio V. (2010). Mitochondrial Energetics and Therapeutics. Annu. Rev. Pathol. Mech. Dis..

[70] Kirillova A., Smitz J.E.J., Sukhikh G.T., Mazunin I. (2021). The Role of Mitochondria in Oocyte Maturation. Cells.

[71] Sousa J.S., D’Imprima E., Vonck J. (2018). Mitochondrial Respiratory Chain Complexes.

[72] Schofield J.H., Schafer Z.T. (2021). Mitochondrial Reactive Oxygen Species and Mitophagy: A Complex and Nuanced Relationship. Antioxid. Redox Signal.

[73] Qi L., Chen X., Wang J., Lv B., Zhang J., Ni B., Xue Z. (2019). Mitochondria: The Panacea to Improve Oocyte Quality?. Ann. Transl. Med..

[74] Aitken R.J., Whiting S., De Iuliis G.N., McClymont S., Mitchell L.A., Baker M.A. (2012). Electrophilic Aldehydes Generated by Sperm Metabolism Activate Mitochondrial Reactive Oxygen Species Generation and Apoptosis by Targeting Succinate Dehydrogenase. J. Biol. Chem..

[75] Aitken R.J., Gibb Z., Baker M.A., Drevet J., Gharagozloo P. (2016). Causes and Consequences of Oxidative Stress in Spermatozoa. Reprod. Fertil. Dev..

[76] Koppers A.J., De Iuliis G.N., Finnie J.M., McLaughlin E.A., Aitken R.J. (2008). Significance of Mitochondrial Reactive Oxygen Species in the Generation of Oxidative Stress in Spermatozoa. J. Clin. Endocrinol. Metab..

[77] Soares M., Sousa A.P., Fernandes R., Ferreira A.F., Almeida-Santos T., Ramalho-Santos J. (2020). Aging-Related Mitochondrial Alterations in Bovine Oocytes. Theriogenology.

[78] Aitken R.J. (2020). Impact of Oxidative Stress on Male and Female Germ Cells: Implications for Fertility. Reproduction.

[79] Showell M.G., Brown J., Clarke J., Hart R.J. (2013). Antioxidants for Female Subfertility. Cochrane Database Syst. Rev..

[80] Ishigami M. (1998). Superoxide Dismutase. Nippon rinsho. Jpn. J. Clin. Med..

[81] Sies H. (2020). Oxidative Stress: Concept and Some Practical Aspects. Antioxidants.

[82] Paul C., Teng S., Saunders P.T.K. (2009). A Single, Mild, Transient Scrotal Heat Stress Causes Hypoxia and Oxidative Stress in Mouse Testes, Which Induces Germ Cell Death. Biol. Reprod..

[83] Marques M., Sousa A.P., Paiva A., Almeida-Santos T., Ramalho-Santos J. (2014). Low Amounts of Mitochondrial Reactive Oxygen Species Define Human Sperm Quality. Reproduction.

[84] Nakai A., Fukushima Y., Yamamoto A., Amatsu Y., Chen X., Nishigori M., Yoshioka Y., Kaneko M., Koshiba T., Watanabe T. (2024). Increased ROS Levels in Mitochondrial Outer Membrane Protein Mul1-deficient Oocytes Result in Abnormal Preimplantation Embryogenesis. FEBS Lett..

[85] Turner T.T., Lysiak J.J. (2008). Oxidative Stress: A Common Factor in Testicular Dysfunction. J. Androl..

[86] O’Flaherty C., Scarlata E. (2022). Oxidative Stress and Reproductive Function: The Protection of Mammalian Spermatozoa against Oxidative Stress. Reproduction.

[87] Neha K., Haider M.R., Pathak A., Yar M.S. (2019). Medicinal Prospects of Antioxidants: A Review. Eur. J. Med. Chem..

[88] Burton G.J., Hempstock J., Jauniaux E. (2003). Oxygen, Early Embryonic Metabolism and Free Radical-Mediated Embryopathies. Reprod. Biomed. Online.

[89] Grimm A., Eckert A. (2017). Brain Aging and Neurodegeneration: From a Mitochondrial Point of View. J. Neurochem..

[90] Smith R.A.J., Hartley R.C., Cochemé H.M., Murphy M.P. (2012). Mitochondrial Pharmacology. Trends Pharmacol. Sci..

[91] Pagano G., Aiello Talamanca A., Castello G., Cordero M.D., D’Ischia M., Gadaleta M.N., Pallardó F.V., Petrović S., Tiano L., Zatterale A. (2014). Oxidative Stress and Mitochondrial Dysfunction across Broad-Ranging Pathologies: Toward Mitochondria-Targeted Clinical Strategies. Oxidative Med. Cell. Longev..

[92] Ogata K., Imai A., Sato S., Nishino K., Watanabe S., Somfai T., Kobayashi E., Takeda K. (2022). Effects of Reduced Glutathione Supplementation in Semen Freezing Extender on Frozen-Thawed Bull Semen and in Vitro Fertilization. J. Reprod. Dev..

[93] Adeoye O., Olawumi J., Opeyemi A., Christiania O. (2018). Review on the Role of Glutathione on Oxidative Stress and Infertility. J. Bras. Reprod. Assist..

[94] Pereira R.M., Baptista M.C., Vasques M.I., Horta A.E.M., Portugal P.V., Bessa R.J.B., e Silva J.C., Pereira M.S., Marques C.C. (2007). Cryosurvival of Bovine Blastocysts Is Enhanced by Culture with Trans-10 Cis-12 Conjugated Linoleic Acid (10t,12c CLA). Anim. Reprod. Sci..

[95] Hu J.-H., Zhao X.-L., Tian W.-Q., Zan L.-S., Li Q.-W. (2011). Effects of Vitamin E Supplementation in the Extender on Frozen-Thawed Bovine Semen Preservation. Animal.

[96] Arkoun B., Galas L., Dumont L., Rives A., Saulnier J., Delessard M., Rondanino C., Rives N. (2019). Vitamin E but Not GSH Decreases Reactive Oxygen Species Accumulation and Enhances Sperm Production during In Vitro Maturation of Frozen-Thawed Prepubertal Mouse Testicular Tissue. Int. J. Mol. Sci..

[97] Chavarria D., Silva T., MagalhãesE Silva D., Remiaõ F., Borges F. (2016). Lessons from Black Pepper: Piperine and Derivatives Thereof. Expert. Opin. Ther. Pat..

[98] Tripathi A.K., Ray A.K., Mishra S.K. (2022). Molecular and Pharmacological Aspects of Piperine as a Potential Molecule for Disease Prevention and Management: Evidence from Clinical Trials. Beni Suef Univ. J. Basic. Appl. Sci..

[99] Lim J.S., Lee D.Y., Lim J.H., Oh W.K., Park J.T., Park S.C., Cho K.A. (2022). Piperine: An Anticancer and Senostatic Drug. Front. Biosci.-Landmark.

[100] Ferreira F., Oliveira A., Lindon F., Oliveira P., Teixeira J., Pereira R.M.N.L. Effect of the Natural Antioxidant Piperine on Maturation of Bovine Oocyte Production and Embryo Production. Proceedings of the XIV Congresso Ibérico Sobre Recursos Genéticos Animais.

[101] Tauskela J.S. (2007). MitoQ—A Mitochondria-Targeted Antioxidant. IDrugs.

[102] Teixeira J., Oliveira C., Amorim R., Cagide F., Garrido J., Ribeiro J.A., Pereira C.M., Silva A.F., Andrade P.B., Oliveira P.J. (2017). Development of Hydroxybenzoic-Based Platforms as a Solution to Deliver Dietary Antioxidants to Mitochondria. Sci. Rep..

[103] Marei W.F.A., Van Den Bosch L., Pintelon I., Mohey-Elsaeed O., Bols P.E.J., Leroy J.L.M.R. (2019). Mitochondria-Targeted Therapy Rescues Development and Quality of Embryos Derived from Oocytes Matured under Oxidative Stress Conditions: A Bovine in Vitro Model. Hum. Reprod..

[104] Elkhawagah A.R., Donato G.G., Poletto M., Martino N.A., Vincenti L., Conti L., Necchi D., Nervo T. (2024). Effect of Mitoquinone on Sperm Quality of Cryopreserved Stallion Semen. J. Equine Vet. Sci..

[105] Teixeira J., Cagide F., Benfeito S., Soares P., Garrido J., Baldeiras I., Ribeiro J.A., Pereira C.M., Silva A.F., Andrade P.B. (2017). Development of a Mitochondriotropic Antioxidant Based on Caffeic Acid: Proof of Concept on Cellular and Mitochondrial Oxidative Stress Models. J. Med. Chem..

[106] Niki E. (1987). Interaction of Ascorbate and A-Tocopherol. Ann. N. Y. Acad. Sci..

[107] Yang X., Zhou D., Gao L., Wang Y., Wang Y., Jia R., Bai Y., Shi D., Lu F. (2024). Effects of Astaxanthin on the Physiological State of Porcine Ovarian Granulose Cells Cultured In Vitro. Antioxidants.

[108] Rostami S., Alyasin A., Saedi M., Nekoonam S., Khodarahmian M., Moeini A., Amidi F. (2023). Astaxanthin Ameliorates Inflammation, Oxidative Stress, and Reproductive Outcomes in Endometriosis Patients Undergoing Assisted Reproduction: A Randomized, Triple-Blind Placebo-Controlled Clinical Trial. Front. Endocrinol..

[109] Oliveira C., Cagide F., Teixeira J., Amorim R., Sequeira L., Mesiti F., Silva T., Garrido J., Remião F., Vilar S. (2018). Hydroxybenzoic Acid Derivatives as Dual-Target Ligands: Mitochondriotropic Antioxidants and Cholinesterase Inhibitors. Front. Chem..

[110] Benfeito S., Oliveira C., Fernandes C., Cagide F., Teixeira J., Amorim R., Garrido J., Martins C., Sarmento B., Silva R. (2019). Fine-Tuning the Neuroprotective and Blood-Brain Barrier Permeability Profile of Multi-Target Agents Designed to Prevent Progressive Mitochondrial Dysfunction. Eur. J. Med. Chem..

[111] Teixeira J., Basit F., Willems P.H.G.M., Wagenaars J.A., van de Westerlo E., Amorim R., Cagide F., Benfeito S., Oliveira C., Borges F. (2021). Mitochondria-Targeted Phenolic Antioxidants Induce ROS-Protective Pathways in Primary Human Skin Fibroblasts. Free Radic. Biol. Med..

[112] Lourenço B. (2020). Effect of Mitochondriotropic Molecules in Reducing Oxidative Stress in Assisted Reprodution Techniques (Tese de Mestrado). Master’s Thesis.

[113] Lourenço B., Ferreira F., Marques C.C., Batista M.C., Teixeira J., Cagide F., Borges F., Oliveira P., Pereira R.M.L.N. Natural Derived Mitochondriotropic Molecules Improve Embryo Quality and Cryosurvival (Poster). Proceedings of the XIII Congreso Ibérico Serga/Sprega Sobre Recursos Genéticos Animales.

[114] Horowitz S. (2009). Coenzyme Q10 One Antioxidant, Many Promising Applications. Altern. Complement. Ther..

[115] Ernster L., Forsmark P., Nordenbrand1 K. (1992). The Mode of Action of Lipid-Soluble Antioxidants in Biological Membranes. Relationship between the Effects of Ubiquinol and Vitamin E as Inhibitors of Lipid Peroxidation in Submitochondrial Particles. J. Nutr. Sci. Vitaminol..

[116] Yildirim R.M., Seli E. (2024). Mitochondria as Therapeutic Targets in Assisted Reproduction. Hum. Reprod..

[117] Chen P., Chen F., Lei J., Li Q., Zhou B. (2019). Activation of the MiR-34a-Mediated SIRT1/MTOR Signaling Pathway by Urolithin A Attenuates d-Galactose-Induced Brain Aging in Mice. Neurotherapeutics.

[118] D’Amico D., Andreux P.A., Valdés P., Singh A., Rinsch C., Auwerx J. (2021). Impact of the Natural Compound Urolithin A on Health, Disease, and Aging. Trends Mol. Med..

[119] Amorim R., Cagide F., Tavares L.C., Simões R.F., Soares P., Benfeito S., Baldeiras I., Jones J.G., Borges F., Oliveira P.J. (2022). Mitochondriotropic Antioxidant Based on Caffeic Acid AntiOxCIN4 Activates Nrf2-Dependent Antioxidant Defenses and Quality Control Mechanisms to Antagonize Oxidative Stress-Induced Cell Damage. Free Radic. Biol. Med..

[120] Fernandes C., Videira A.J.C., Veloso C.D., Benfeito S., Soares P., Martins J.D., Gonçalves B., Duarte J.F.S., Santos A.M.S., Oliveira P.J. (2021). Cytotoxicity and Mitochondrial Effects of Phenolic and Quinone-based Mitochondria-targeted and Untargeted Antioxidants on Human Neuronal and Hepatic Cell Lines: A Comparative Analysis. Biomolecules.

[121] Teixeira C., Marques C.C., Baptista M.C., Pimenta J., Teixeira J., Cagide F., Borges F., Montezinho L., Oliveira P., Pereira R.M.L.N. (2020). Beneficial Effect of Mitochondriotropic Antioxidants on Oocyte Maturation and Embryo Production. Eur. J. Clin. Investig..

[122] Ferreira F.C., Sousa A., Marques C.C., Baptista M.C., Teixeira J., Cagide F., Borges F., Oliveira P., Pereira R.M.L.N. Effect of a Mitochondriotropic Antioxidant Based on Caffeic Acid (AntiOxCIN4) on Spermatozoa Capacitation and in Vitro Fertilization (Poster). Proceedings of the Congresso CIISA “Inovação em Pesquisa Animal, Veterinária e Biomédica”.

[123] Leopoldini M., Russo N., Toscano M. (2011). The Molecular Basis of Working Mechanism of Natural Polyphenolic Antioxidants. Food Chem..

[124] Manach C., Scalbert A., Morand C., Rémésy C., Jiménez L. (2004). Polyphenols: Food Sources and Bioavailability. Am. J. Clin. Nutr..

[125] Li Y.R., Li S., Lin C.C. (2018). Effect of Resveratrol and Pterostilbene on Aging and Longevity. BioFactors.

[126] Oroian M., Escriche I. (2015). Antioxidants: Characterization, Natural Sources, Extraction and Analysis. Food Res. Int..

[127] Shirzeyli M.H., Amidi F., Shamsara M., Nazarian H., Eini F., Shirzeyli F.H., Zolbin M.M., Novin M.G., Joupari M.D. (2020). Exposing Mouse Oocytes to Mitoq during in Vitro Maturation Improves Maturation and Developmental Competence. Iran. J. Biotechnol..

[128] Câmara D.R., Ibanescu I., Siuda M., Bollwein H. (2022). Mitoquinone Does Not Improve Sperm Cryo-resistance in Bulls. Reprod. Domest. Anim..

